# Retraction: Improved Cellular Specificity of Plasmonic Nanobubbles versus Nanoparticles in Heterogeneous Cell Systems

**DOI:** 10.1371/journal.pone.0187820

**Published:** 2017-11-02

**Authors:** The PLOS ONE Editors

Following publication, concerns were raised about similarities between two figures in this *PLOS ONE* publication [1] and images published by the same corresponding author in the *Journal of Surgical Research* [2] and *ACS Nano* [3].

- *PLOS ONE* Figure 3c [1], is similar to *Journal of Surgical Research* Figure 4a [2] and *ACS Nano* Figure 4c [3]. The voltage values in *PLOS ONE* Figure 3c [1] and *Journal of Surgical Research* Figure 4a [2] are identical if the time coordinates are offset by 6 ns. Comparing *PLOS ONE* Figure 3c [1] and *ACS Nano* Figure 4c [3], the traces appear identical except in the 0–25 ns interval, and the unit labels on both x- and y-axes differ between the two figures.
- *PLOS ONE* Figure 3d [1] is similar to the original published version of *Journal of Surgical Research* Figure 4d [2]. The traces are identical when offset on the x-axis and scaled in the y-axis, and the trace appears to include a segment that is repeated three times within each figure.

The authors noted that errors were made during preparation of the figures in the *PLOS ONE* article, and claim that both sets of figures were used in these articles as illustrative examples of the presence or absence of a single plasmonic nanobubble event by showing typical individual time-responses. An erratum was published in the *Journal of Surgical Research* in 2015, providing a new image for Figure 4d [4].

The Rice University Office of Research investigated these concerns and concluded that there was evidence of data falsification involving Figures 3c and 3d of the *PLOS ONE* article. Due to the concerns about image duplication and data falsification, and in line with Rice University’s recommendation, the *PLOS ONE* Editors retract this article.
